# [18F]FDDNP performed better than [18F] Florbetapir to distinguish transthyretin cardiac amyloidosis (TTR-CA) patients from healthy controls: an ex vivo study

**DOI:** 10.1186/1750-1172-10-S1-P38

**Published:** 2015-11-02

**Authors:** Anne-Claire Dupont, Denis Guilloteau, Rana Ben-Azzouna, Rudy Bidault, Vincent Algalarrondo, Michel Slama, Nicolas Arlicot, Dominique Le Guludec

**Affiliations:** 1 CHRU TOURS, Nuclear medicine, 37000, Tours, France; 2 INSERM, U698, 78000, Paris, France; 3 INSERM, U930, 37000, Tours, France; 4 AP-HP, Nuclear medicine, 78000, Paris, France

## Background

TTR-CA is characterized by extracellular depositions of amyloid β (Aβ) which can lead to arrhythmias, heart failure and even sudden death. While early diagnosis of TTR-CA has important therapeutic and prognostic impact, there is no sensitive and quantitative tool to document the location and extent of cardiac Aβ in these patients. So we aimed to test and compare the usefulness for TTR-CA early diagnosis of both [18F]florbetapir and [18F]FDDNP, 2 PET tracers validated for brain detection of Aβ.

## Methods

Binding of both radiopharmaceuticals to Aβ was evaluated in myocardial tissue from patients who underwent cardiac transplantation either for TTR-CA, or ischemic heart failure as control. Heart sections were incubated with [18F]florbetapir or [18F]FDDNP at concentration of 3nM. Nonspecific binding was assessed by incubation of adjacent sections in the presence of an excess of cold ligand. Autoradiograms were treated with a grey-level analysis method. Regions of interest were delimited and the modal grey value were determined.

## Results

[18F]FDDNP uptake in TTR-CA myocardial sections (nP=6) was significantly higher (+86%) compared to controls (nT=3) whereas no significant difference was observed with [18F]florbetapir (nP=4 and nT=2, +32%, p=0.13). The mean ratio (specific binding patient/specific binding controls) were 11.9±2.0 for [18F]FDDNP and 1.5±0.1 for [18F]florbetapir (comparison : p=0.01). Nevertheless, the intensity of both radiotracers binding strongly decreased in sections with unlabeled ligand (-74%, and -83 respectively), suggesting Aβ specificity.

## Conclusion

[18F]FDDNP and [18F]florbetapir, are able to bind ex vivo specifically to Aβ in heart tissue. The largely improved ratio of specific binding (patient/controls) of [18F]FDDNP, compared to [18F]florbetapir, strongly suggests its better sensibility and then diagnostic potential to discriminate in vivo ATTR patients from healthy subjects.

